# Solid-State Fermentation with *Aspergillus niger* GH1 to Enhance Polyphenolic Content and Antioxidative Activity of Castilla Rose (*Purshia plicata*)

**DOI:** 10.3390/plants9111518

**Published:** 2020-11-09

**Authors:** De León-Medina José Carlos, Sepúlveda Leonardo, Morlett-Chávez Jesús, Meléndez-Renteria Paola, Zugasti-Cruz Alejandro, Ascacio-Valdés Juan, Aguilar Cristóbal Noé

**Affiliations:** 1 Bioprocesses and Bioproducts Research Group, Food Research Department, School of Chemistry, Autonomous University of Coahuila, Saltillo 25280, Mexico; jose.deleon@uadec.edu.mx (D.L.-M.J.C.); leonardo_sepulveda@uadec.edu.mx (S.L.); cristobal.aguilar@uadec.edu.mx (A.C.N.); 2 Laboratory of Molecular Biology, School of Chemistry, Autonomous University of Coahuila, Saltillo 25280, Mexico; antoniomorlett@uadec.edu.mx; 3 Research and Conservation Center of Coahuila Biodiversity and Ecology, Autonomous University of Coahuila, Cuatrociénegas 27640, Mexico; paola.melendez@uadec.edu.mx; 4 Laboratory of Toxicology, School of Chemistry, Autonomous University of Coahuila, Saltillo 25280, Mexico; alejandro_zugasti@uadec.edu.mx

**Keywords:** antioxidant activity, *Aspergillus niger* GH1, Castilla Rose, polyphenols, solid-state fermentation

## Abstract

This work was performed to study Castilla Rose (*Purshia plicata*) as a potential source of polyphenols obtained by solid-state fermentation (SSF)-assisted extraction using the microorganism *Aspergillus niger* GH1 and to evaluate the antioxidant activity of the extracted compounds. First, water absorption capacity (WAC) of the plant material, radial growth of the microorganism, determination of best fermentation conditions, and maximum accumulation time of polyphenols were tested. Then, a larger-scale fermentation, polyphenols isolation by column liquid chromatography (Amberlite XAD-16) and recovered compounds identification by HPLC-MS were made. Finally, the antioxidant activity of the recovered compounds was tested by ABTS, DPPH, and lipid oxidation inhibition assays. The best fermentation conditions were temperature 25 °C and inoculum 2 × 10^6^ spores/g, while the maximum extraction time of polyphenols was 24 h (173.95 mg/g). The HPLC/MS analysis allowed the identification of 25 different polyphenolic compounds, and the antioxidant activity of the obtained polyphenols was demonstrated, showing ABTS assay the most effective with inhibition of 94.34%.

## 1. Introduction

Castilla Rose (*Purshia plicata*) is a plant that belongs to the family *Rosaceae,* a native of the semi-desert area located in northeast Mexico, mainly in Coahuila, Nuevo Leon and Chihuahua states [1]. There is scarce information about this plant’s properties; still, it has been used in traditional Mexican medicine as a resource for stomach diseases and other afflictions, also this plant can be used as an ingredient for some food preparations, so it might be considered a potential source of phytochemicals, like polyphenolic compounds.

Polyphenolic compounds are of vegetal origin and can be present in diverse plant parts such as leaves, stem, or bark and are stored in cell vacuoles [2]. They have a molecular weight of 500 to 3000 Da approximately, most of them are water-soluble, can bind the proteins and precipitate them, and possess and astringent flavor [3]. They are considered the main secondary metabolites of plants [4]. The classification of these compounds is divided mainly into two big groups that are hydrolysable and condensed polyphenols; however, recently, their classification has been reported in four groups that are ellagitannins, gallotannins, condensed tannins and complex tannins [5]. These compounds can be ingested in diverse food sources: berries like strawberries, cranberries and blackberries are the foods group with the highest polyphenolic compounds content [6].

Similarly, Aguilera-Carbó et al. (2008) reported that some plants of the Mexican semi-desert, such ascreosote bush (*Larrea tridentata*), dragon’s blood (*Jatropha dioca*), hojasen (*Flourensia cernua*), candelilla (*Euphorbia antisyphilitica*) and pomegranate (*Punica granatum*) are excellent sources for the recovery of polyphenolic compounds [7]. Some studies have shown that these compounds have several interesting biological activities, including antimicrobial, antioxidant and anticancer [8,9,10]. Due to this, the interest in the study and design of strategies for the extraction and recovery of these compounds has been increased in recent years. In this sense, extraction techniques known as conventional have been developed with the use of organic solvents like methanol or acetone; however, the obtained yields are generally low, and there is a high production of contaminant wastes [11]. For that reason, one of the alternatives to extract these kinds of compounds by the use of green technologies, which generates a lower amount of contaminant wastes, is the use of bioprocesses, specifically, solid-state fermentation.

Solid-state fermentation (SSF) can be defined as the fermentative process in which the microorganisms grow in solid materials without free water [12]. In this process are used microorganisms, mainly filamentous fungi, due to their ability to grow on the support/substrate used. It has been also demonstrated that bioactive compounds such as polyphenols can be extracted by this bioprocess using filamentous fungi, like *Aspergillus niger* GH1 [13].

The aim of this work was to extract, recover and identify polyphenolic compounds of Castilla Rose (*Purshia plicata*) by solid-state fermentation-assisted extraction using the filamentous fungi *Aspergillus niger* GH1.

## 2. Materials and Methods

### 2.1. Plant Material

Castilla Rose plant was collected from La Angostura, Saltillo, Coahuila, Mexico. The plant was dehydrated at 50 °C for 72 h, then leaves and flowers were removed manually and stored in plastic bags protected from light at room temperature.

### 2.2. Microorganism and Culture Medium

The strain *Aspergillus niger* GH1 (Food Research Department Collection, Autonomous University of Coahuila, Mexico) was used in this study. The strain was previously conserved in a cryoprotective solution (skimmed milk/glycerol 9:1) at −55 °C. The strain was activated in potato dextrose agar (PDA-Bioxon) for 5 days at 30 °C. The spores were recovered using 0.01% Tween 80, and spores/mL were calculated by counting in Neubauer’s chamber.

### 2.3. Characterization of Plant Material

#### 2.3.1. Water Absorption Capacity and Maximum Moisture

Raw material (1.25 g) was placed in 50 mL centrifuge tubes, 30 mL of distilled water was added, the sample was incubated at 30 °C and stirred 10 min after the heating started. Then the sample was centrifuged at 4900 rpm for 30 min, the supernatant was discarded, the remaining gel was weighed, and water absorption capacity (WAC) was calculated and expressed as gel grams per grams of dry weight (gel *g/g*). Moisture content and solids present in the material was determined by weighing 0.55 g in a thermobalance (OHAUS, Parsippany, NJ, USA). Using WAC values, solids and moisture content, the maximum material moisture was calculated.

#### 2.3.2. Radial Growth of *Aspergillus niger* GH1 on Plant Material

The plant material (3 g) was placed in a Petri plate, and 7 mL of distilled water was added (moisture 70%). *Aspergillus niger* GH1 was inoculated at the center of the plate and was incubated at 30 °C. Mycelial growth was measured every 6 h until the complete invasion of the plate.

### 2.4. Determination of Best Fermentation Conditions

In order to establish the best fermentation conditions, an exploratory evaluation was carried out at 60 h using a Box Hunter & Hunter design using the software Statistica 7.0. The evaluated conditions were inoculum and temperature with maximum and minimum levels recorded for each one. The response variable measured was total polyphenols (obtained by summation of hydrolysable and condensed polyphenols). The treatments are shown in Table 1.

Solid-state fermentation was carried out in Erlenmeyer flasks (250 mL) with 3 g of Castilla Rose and 7 mL of Czapeck–dox culture media with the following components (g/L): NaNO_3_ (7.65), KH_2_PO_4_ (3.04), MgSO_4_·7H_2_O (1.52) and KCl (1.52) with the corresponding *Aspergillus niger* GH1 inoculum (moisture 70%). The samples were incubated with the corresponding temperature for 60 h. The extracts recovery was carried out by distilled water addition (14 mL) and subsequent filtration. The recovered extracts were stored at −20 °C and protected from light until the determination of total polyphenolic content to establish its maximum accumulation time.

### 2.5. Determination of Hydrolyzable Polyphenols

Hydrolyzable polyphenols present in fermentation extracts of Castilla Rose were determined using Folin–Ciocalteu’s reagent (Sigma-Aldrich, México City, Mexico). The test was done by triplicate and the results were expressed asgallic acid equivalents [14].

### 2.6. Determination of Condensed Polyphenols

Condensed polyphenols present in fermentation extracts of Castilla Rose were determined using ferric reagent and HCl-butanol (1:9). The test was done by triplicate and the results were expressed as equivalents of catechin [15].

### 2.7. Higher Volume Fermentation and Polyphenolic Compounds Recovery

Once maximum accumulation time was established, a higher-volume fermentation was carried out to recover an adequate quantity of polyphenolic compounds. This fermentation was performed using 500 g of Castilla Rose in a plastic tray reactor, then 1167 mL of Czapeck–dox culture media inoculated with *Aspergillus niger* GH1 (2 × 10^6^ esp/g) was added. Moisture was 70%. The fermentation was carried out at 25 °C for 24 h, extract recovery was made adding 2334 mL of distilled water and then the extract was filtered. The polyphenolic compounds present in the extract were separated and recovered by column liquid chromatography using Amberlite XAD-16 [16]. First, an elution with water was done to remove not relevant compounds and then an elution with ethanol was done to obtain the polyphenolic fraction. The ethanol was evaporated and the compounds were recovered as a fine powder.

### 2.8. RP-HPLC-ESI-MS Analysis

The analyses by reverse phase-high performance liquid chromatography were performed on a Varian HPLC system, including an autosampler (VarianProStar 410, Palo Alto, CA, USA), a ternary pump (VarianProStar 230I, Palo Alto, CA, USA) and a PDA detector (VarianProStar 330, Palo Alto, CA, USA). A liquid chromatograph ion trap mass spectrometer (Varian 500-MS IT Mass Spectrometer, Palo Alto, CA, USA) equipped with an electrospray ion source also was used. Samples (5 µL) were injected onto a Denali C18 column (150 × 2.1 mm, 3 µm, Grace, Palo Alto, CA, USA). The oven temperature was maintained at 30 °C. The eluents were formic acid (0.2%, *v/v*; solvent A) and acetonitrile (solvent B). The following gradient was applied: initial, 3% B; 0–5 min, 9% B linear; 5–15 min, 16% B linear; 15–45 min, 50% B linear. The column was then washed and reconditioned. The flow rate was maintained at 0.2 mL/min, and elution was monitored at 245, 280, 320 and 550 nm. The whole effluent (0.2 mL/min) was injected into the source of the mass spectrometer, without splitting. All MS experiments were carried out in the negative mode [M-H]^−1^. Nitrogen was used as nebulizing gas and helium as damping gas. The ion source parameters were: spray voltage 5.0 kV, and capillary voltage and temperature were 90.0 V and 350 °C, respectively. Data were collected and processed using MS Workstation software (V 6.9). Samples were firstly analyzed in full scan mode acquired in the *m/z* range 50–2000 [17].

### 2.9. Antioxidant Activity

#### 2.9.1. ABTS Antioxidant Assay

The 2,2′-Azino-bis(3-Ethylbenzothiazoline-6-Sulfonic Acid) (ABTS) free radical was prepared by mixing potassium persulfate 2.45 mM with ABTS solution 7 mM (1:2 *v/v*). The mixture was set in a dark room for 12–16 h. After this time, the absorbance of the solution was measured at 734 nm and was adjusted at 0.700 by the addition of ethanol. The reaction was carried out by mixing 10 µL of the sample (Castilla Rose polyphenols) and 1 mL of ABTS adjusted solution; then, the absorbance was measured at 734 nm. Water was used as a control, and the results were calculated by the following Equation (1) and reported as inhibition percentage [18] (all experiments were carried out in triplicate):

(1) $$\%\;\mathrm{inhibition}\;=\;\left[1-\left(\frac{A_{sample}}{A_{control}}\right)\right]\times 100\%$$

where *A* = absorbance (nm).

#### 2.9.2. DPPH Antioxidant Assay

The 2,2-diphenyl-1-picrylhidracyl (DPPH) free radical was prepared at 60 µM concentration in methanol. Then, 193 µL of DPPH-methanol solution was mixed with 7 µL of the sample (Castilla Rose polyphenols) and the solution was allowed to stand for 30 min at room temperature. The absorbance of solutions was determined by a microplate reader at 517 nm (TECAN Sunrise, Männedorf, Switzerland). DPPH-methanol was used as a control. The results were reported as the inhibition percentage using Equation (1) [19] (all experiments were carried out in triplicate).

#### 2.9.3. Lipid Oxidation Inhibition Assay

Linoleic acid was employed as the lipid source. The solution was prepared with 0.35 g of linoleic acid and 0.937 g of Tween 20 in 5 mL of 96% ethanol. The reaction was carried out mixing 50 µL of the sample (Castilla Rose polyphenols), 100 µL of linoleic acid solution and 1.5 mL acetate buffer 0.02 M pH 4.0. The mixture was homogenized and incubated for 1 min at 37 °C. Then, 750 µL of FeCl_2_-EDTA0.5 mM was added to induce the oxidation and the samples were incubated for 1 h and 24 h at 37 °C. After each incubation time, 250 µL of the solution was mixed with 1 mL of NaOH 0.1 Min 10% ethanol, and then 2.5 mL of ethanol 10% was added. The absorbance was measured at 232 nm using ethanol 10% as blank. Antioxidant activity percentage was calculated using the Equation (2):

(2) $$\%\;\;LOI=\;\left(\frac{\Delta_{Dcontrol}-\Delta_{Dsample}}{\Delta_{Dcontrol}}\right)\times 100$$

where Δ*_Dcontrol_* is the difference between the absorbance of the control (distilled water) after 24 h and 1 h of incubation and Δ*_Dsample_* is the difference between the absorbance of the sample after 24 and 1 h of incubation [20] (all experiments were carried out in triplicate).

## 3. Results

### 3.1. Water Absorption Capacity

The results of moisture and solids of the dry plant are shown in Table 2. Additionally, the maximum moisture that the plant can accumulate was measured, and it was 83%, while the value of the water absorption capacity obtained for Castilla Rose was 5.3 g gel/g dry weight.

### 3.2. Radial Growth of Aspergillus niger GH1 on Plan Material

**Figure 1:**
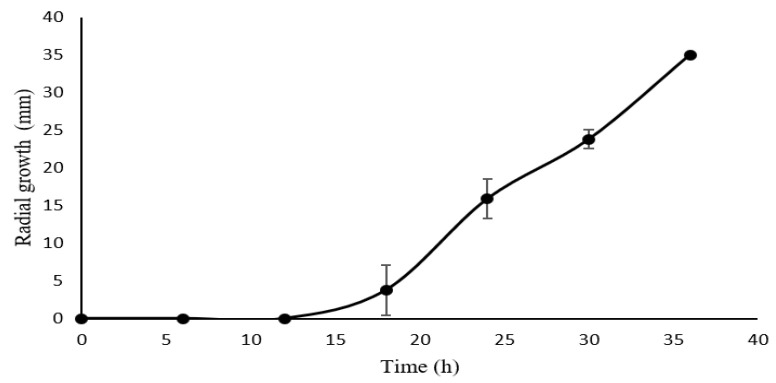
Kinetic of radial growth of *Aspergillus niger* GH1 on Castilla Rose.

In Figure 1, the results of radial growth are shown. It can be observed that *Aspergillus niger* GH1 used the first 6 h to adapt to the substrate, but after the 12 h, its growth was constant until the complete invasion of the Petri plate at 36 h.

### 3.3. Determination of Best Fermentation Conditions

**Figure 2:**
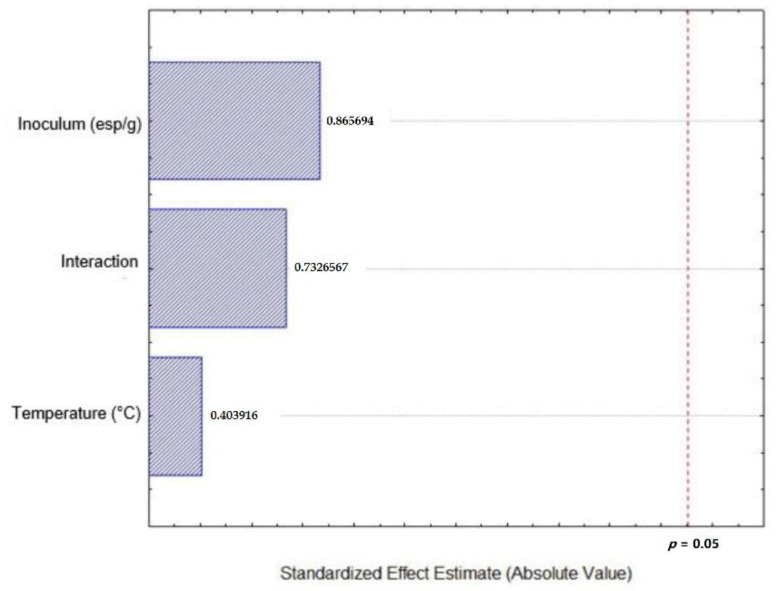
Pareto chart for total polyphenols from the Box Hunter &Hunter design analysis.

Figure 2 shows the Pareto chart where are exposed the evaluated factors in the Box Hunter & Hunter experimental design, temperature, inoculum and their interaction. It can be observed that none of these factors exceeds the confidence line of 95%, meaning that none of the factors have a significant effect on the extraction of total polyphenols from Castilla Rose.

**Figure 3:**
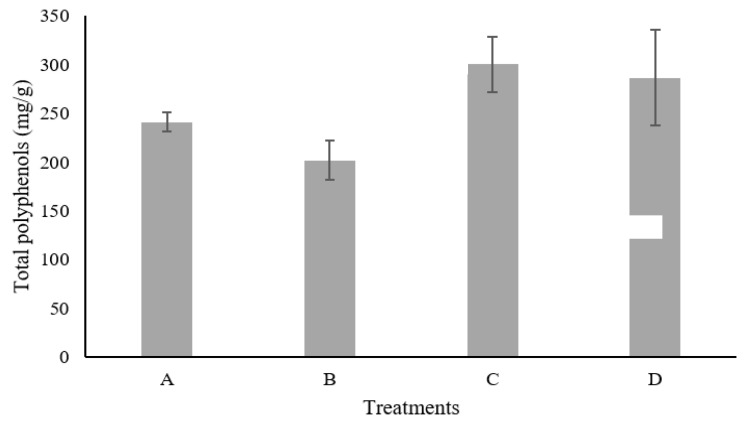
Extraction of total polyphenols by fermentation of Castilla Rose using *Aspergillus niger* GH1.A (25 °C, 2 × 10^6^ esp/g); B (30 °C, 2 × 10^6^ esp/g); C (25 °C, 2 × 10^7^ esp/g); D (30 °C, 2 × 10^7^ esp/g).

Figure 3 shows the comparison between the different treatments for the total polyphenolic compounds by solid-state fermentation. In treatment A (25 °C, 2 × 10^6^ esp/g), the obtained value of total polyphenolic compounds was 240.85 mg/g; 202.065 mg/g in treatment B (30 °C, 2 × 10^6^ esp/g); 302.007 mg/g in treatment C (25 °C, 2 × 10^7^ esp/g) and 286.425 mg/g in treatment D (30 °C, 2 × 10^7^ esp/g).

The first fermentation was carried out to determine the influence of the factors evaluated on the polyphenolic content, where it was demonstrated that the factors evaluated do not have a significant effect on the response (as observed in the Pareto chart). Based on this, treatment A was selected.

### 3.4. Determination of Maximum Time for the Accumulation of Polyphenolic Compounds

A second fermentation was carried out to know the time where the highest quantity of polyphenolic compounds was accumulated. The results (Table 3) show that the polyphenolic content of the plant before fermentation (at 0 h) was 119.22 ± 8.70 mg/g of dry plant. The time where the maximum quantity of obtained polyphenolic compounds was at 24 h with a value of 173.95 ± 4.72 mg/g of dry plant. So, it can be demonstrated that SSF promotes the extraction of these compounds.

The second fermentation was carried out using the conditions of treatment A and was performed for 60 h, sampling every 24 h. It was determined that the maximum accumulation time of compounds was 24 h (the time for maximum accumulation of polyphenolic compounds is presented). It is important to mention that this work is not an optimization study, it is an exploration study of conditions as mentioned in the methodology for the Box Hunter & Hunter design.

### 3.5. RP-HPLC-ESI-MS Analysis

Once we established the best fermentation conditions (temperature and inoculum) and the maximum accumulation time of polyphenols (24 h), a higher-volume fermentation was done using the conditions mentioned before. Then the recovered extract was treated with Amberlite XAD-16 to recover the polyphenolic compounds. Finally, the compounds were characterized by HPLC-MS. The identified compounds are shown in Table 4.

**Figure 4:**
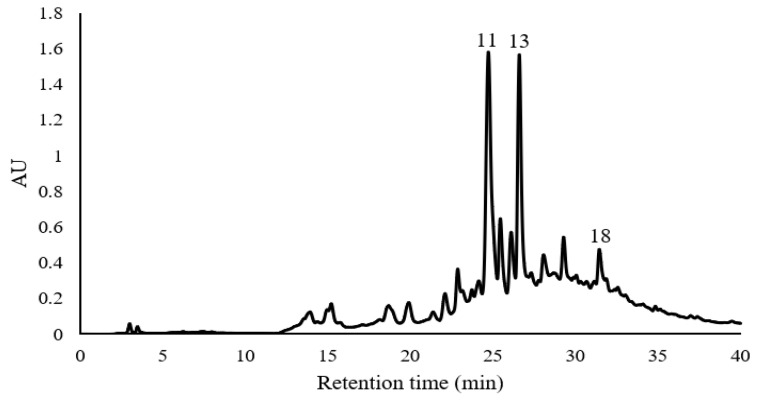
Chromatogram of the recovered polyphenols from Castilla Rose. Compound 11 Kaempferol 3,7-O-diglucoside; compound 13 procyanidin trimer C1; compound 18 ellagic acid.

Figure 4 shows the results obtained by HPLC of the recovered polyphenolic compounds from Castilla Rose fermentation. After doing the RP-HPLC-ESI-MS, it was found that the fermentation extract of Castilla Rose that was treated with Amberlite XAD-16 contained a large number of polyphenolic compounds of great importance, which are shown in Table 5. Ellagic acid, procyanidin C1 trimer, and kaempferol 3,7-O-diglucoside were the main compounds present in the sample.

### 3.6. Antioxidant Activity

The polyphenols recovered from SSF of Castilla Rose demonstrated antioxidant activity by the three assays tested. ABTS assay had the highest inhibition percentage value of 94.34% ± 1.98. DPPH inhibition was 68.71% ± 0.97, and lipid oxidation inhibition was 71.49% ± 1.25 (Table 6). For the antioxidant activity tests the liquid fraction obtained by Amberlite XAD-16 chromatography was used.

## 4. Discussion

The water absorption capacity (WAC) determines how the substrate can be bound to water molecules by the availability of its hydrophilic groups, and the interaction of the water with macromolecules allows the formation of the gel [21]. The substrates that have a high WAC are more suitable to perform a solid-state fermentation because the microorganism presents a higher growth and development [22]. The Castilla Rose plant presented maximum moisture of 83% (Table 2), which is a value that allows performing a fermentation using it as a support. However, when the microorganism used for the fermentation is a filamentous fungus, it is not appropriate to carry out a fermentation with a high value of moisture, as this can interfere with oxygen transfer, and also can cause agglomeration of particles and bacterial growth [23]. Additionally, with high moisture, there is a risk of having an amount of free water in the system that will affect the process because, in solid-state fermentation, there should not be the presence of free water [24]. According to the factors mentioned above, it was decided to carry out the fermentations with the moisture at 70%, which is a value widely used in SSF with filamentous fungi. To carry out an SSF, it is preferred to have high WAC values [22]. Buenrostro-Figueroa et al. (2014) report the WAC of different supports used in solid-state fermentation. The materials with the highest WAC value were coconut husk (12.09 g gel/g dry weight) and sugarcane bagasse (9.46 g gel/g dry weight). The obtained value of Castilla Rose WAC (5.37 g gel/g dry weight) was lower than those of the mentioned supports; however, the value of WAC of Castilla Rose was higher than those of the other two supports used, such as candelilla stalks (3.14 g gel/g dry weight) and corn cobs (2.97 g gel /g dry weight). So, it is demonstrated that the value of the Castilla Rose’s WAC is within the values obtained from other supports used before in SSF. This value allows us to carry out an SSF [25].

Some supports used in solid-state fermentation provide the carbon source for the development of microorganisms and allow it to invade the surface and grow [26]. Filamentous fungi are the most employed microorganisms in solid-state fermentation because their hyphae can grow on the substrate surface and can penetrate the intra-individual spaces and colonize them [27]. Cruz-Hernández et al. (2005) have reported that *Aspergillus niger* GH1 was isolated from plants of Mexican desert with high polyphenolic content such as *Larrea tridentata, Pinus cembroides*, *Carya illioensis,* and *Quercus* spp. Additionally, it has been proved that this strain can grow in culture mediums with minimum requirements, which makes it an interesting strain for use in a solid-state fermentation-assisted extraction to obtain the polyphenolic compounds present in Castilla Rose plants [22,28]. Solid-state fermentation using filamentous fungi has been widely reported for the extraction of diverse bioactive compounds, like polyphenolic compounds, due to these kinds of microorganisms producing different enzymes during their growth that allow the bioavailability of the compounds, thus achieving their extraction [29].

In case of the determination of best fermentation conditions, according to the statistical analysis, there was no significant difference between the four evaluated treatments for the extraction of total polyphenolic compounds, so the following experiments were performed employing the conditions of treatment A (25 °C, 2 × 10^6^ spores/g).In a study reported by Sepúlveda et al. (2012), an exploratory design Plackett–Burman using pomegranate peels and *Aspergillus niger* GH1 was made to observe the influence of different factors on total polyphenols extraction by solid-state fermentation where temperature and inoculum were tested. That study showed that temperature (25–30 °C) had a significant effect in the extraction of ellagic acid (a polyphenolic compound); the optimal temperature was 30 °C. However, inoculum (1 × 10^6^–1 × 10^7^ esp/g) had no significant effect on ellagic acid extraction [30]. In this study, using Castilla Rose, a similar behavior was obtained, except for temperature: this factor had no significant effect. In a solid-state fermentation, the temperature is one of the most significant factors to consider because it can affect the production of different metabolites and enzymes; also, the moisture can be affected, and therefore, the fungus activity might be reduced [26]. Filamentous fungi are mesophilic microorganisms, so their temperature range for growth is from 20 to 55 °C; however, the optimal temperature for growth can be different from the optimal temperature to produce metabolites of interest [31].

The maximum accumulation time of polyphenolic compounds in this study was lower than that reported by Martins et al. (2013), where extraction time of polyphenols from *Larrea tridentata*, a semi-desert plant, was 168 h. It is important to mention that to determine the total polyphenolic content, tests for total hydrolysable polyphenols and total condensed polyphenols should be considered, because the Folin−Ciocalteu’s reagent reacts mainly with the galloyl or HHDP groups, while the ferric reagent and hydrolysis with HCl-butanol act on compounds based on flavan-3-ol, which are within the classification of condensed polyphenols. The total polyphenolic content in the present study was higher also. The difference can be due to the microorganism used for fermentation. In the present study was employed *Aspergillus niger* GH1, a strain widely used for the extraction of polyphenolic compounds; meanwhile, Martins et al. used the microorganism *Phanerochaete chrysosporium,* a microorganism that is used for the degradation mainly of lignin [32,33]. These results also can be compared with those obtained by Aguilar et al. (2008), where maximum accumulation time of polyphenolic compounds extracted from pomegranate husk and *Larrea tridentata* leaves by solid-state fermentation using *Aspergillus niger* GH1 was 96 h, which also was higher than the obtained value in this study [13]. In the first fermentation, where the best conditions of the temperature and inoculum were defined, the maximum amount of total polyphenols obtained was 286.42 mg/g at 60 h.

Meanwhile, in the second one, where the time of maximum accumulation of polyphenolic compounds was set, the highest value of total polyphenols was 173.95 mg/g at 24 h. So, it can be noticed that there is a difference in the total polyphenolic content between these two fermentations. This can be due to the determination of condensed polyphenols because there are many factors in the reaction that can affect the quantification, such as the content of water, the number of phenolic groups of the compounds and the color yield. Additionally, the microorganism probably adapted differently to the support/substrate (plant material) in the two fermentations and this caused the differences in the contents of polyphenolic compounds [34].

With the results of the compounds of Castilla Rose identified before and after fermentation, it can be noticed that the number of compounds increased when the fermentation was done, so this demonstrates that solid-state fermentation effectively allows the extraction of these kinds of compounds. The three compounds of more importance presented after fermentation were ellagic acid, procyanidin C1 trimer, and kaempferol 3,7-O-diglucoside.Ellagic acid is a molecule that has been widely studied because it has relevant biological properties (such as antioxidant, antimicrobial anticancer, etc.) against diverse agents that can cause health damage, like free radicals. These agents represent a risk in the formation of cancerogenic tumors, and it has been proved that this compound induces apoptosis in carcinogenic cells [35,36]. Additionally, ellagic acid is a molecule highly thermostable which can be potentially used in the food industry as a nutraceutical [37]. Kaempferol belongs to the flavonols family, and it has been reported that it is present in plant-based foods like broccoli, cabbage, tomato, beans, apples and tea (80%). It has also been reported that this compound affects different pathways related to the unleashing of several adverse clinical-pathologic aspects [38]. Kaempferol also possesses a great antioxidant activity and can react with substances like H_2_O_2_, HOCl, superoxide, nitric oxide, etc. [39]. Procyanidins are polyphenolic compounds present in plants and belong to the flavonols family also; the most common are derivates of epicatechin [40]. These compounds are present in foods like cereals and vegetables and are abundant in some fruits, fruit juices, cocoa, tea and wine. It has been demonstrated that procyanidins have an antioxidant activity 50 times higher than that of other known antioxidants, such as Vitamin C and E [41]. Specifically, procyanidin trimer C1 has been studied in signaling pathways to regulate some inflammatory processes [42].

ABTS assay is based on the reagent 2,20-azino-bis (3-ethylbenzthiazoline-6-sulphonic acid) which is a free radical with an intense coloration, so when an antioxidant agent is bound to this compound, there is a decrease in its concentration and therefore in its coloration, which can be measured in a spectrophotometer [43]. By this method, it is possible to examine the antioxidant activity of hydrophilic and lipophilic compounds [44]. The obtained value in this study (94.34%) shows that almost all free radicals present in the reaction where inhibited. This was similar to the results obtained by Aguirre-Joya et al. (2018), where an inhibition of 97% using polyphenolic compounds from *Larrea tridentata* (a Mexican semi-desert plant) was reported [45]. DPPH assay is similar to ABTS assay, where the free radical 1,1-diphenyl-2-picrylhydrazyl (DPPH) presents an intense coloration that diminishes when it has contact with an antioxidant agent. In this study, the inhibition value obtained for this radical was 68.71%, which is lower than reported for polyphenolic compounds extracted from candelilla (*Euphorbia antisyphilitica*), another Mexican semi-desert plant, which presented an inhibition value of 92.33%. This could be due to the different compounds present in each plant [9].

On the other hand, the lipid oxidation inhibition assay is based on the inhibition of the peroxyl radicals by hydrogen atom transfer [46,47]. The results obtained for this assay can be compared to those reported by Aguirre-Joya et al. (2018), where 57% inhibition was obtained using polyphenolic compounds from *Larrea tridentata*, which is lower compared to the obtained value in this work (71.49%) [45]. This confirms that polyphenolic compounds from Castilla Rose were more able to inhibit the peroxyl radicals in vitro.

Castilla Rose plant is a suitable support/substrate to perform an SSF because it presented an adequate water absorption capacity and moisture, and the microorganism *Aspergillus niger* GH1 was able to grow and invade the plant material. The time of maximum accumulation of polyphenolic compounds (24 h) was lower than in other studies. The SSF allowed the extraction and characterization of a total of 25 different compounds, which presented an antioxidant activity in vitro by the assays tested, proving that these compounds have biological activities that can be beneficial for human health and could be used in the food industry as functional ingredients.

## Figures and Tables

**Table 1:** Treatment matrix of experimental design Box Hunter & Hunter.

Treatment	Temperature (°C)	Inoculum (spores/g)
2	30	2 × 10^6^
10	30	2 × 10^6^
9	25	2 × 10^6^
3	25	2 × 10^7^
4	30	2 × 10^7^
5	25	2 × 10^6^
12	30	2 × 10^7^
8	30	2 × 10^7^
1	25	2 × 10^6^
7	25	2 × 10^7^
6	30	2 × 10^6^
11	25	2 × 10^7^
	**Levels**	
**Factor**	+1	−1
**Temperature (°C)**	30	25
**Inoculum (esp/g)**	2 × 10^7^	2 × 10^6^

**Table 2:** Data obtained about water absorption capacity of Castilla Rose (in dry base).

Parameters	Results
Moisture (%)	6.8
Solids (%)	93.2
Water absorption capacity (g of gel/g of dry weight)	5.3
Maximum moisture of the support/substrate (%)	83

**Table 3:** Total polyphenols extracted by sold-state fermentation (SSF) from Castilla Rose.

Fermentation Time	Total Polyphenols (mg/g of Dry Plant)
0 h	119.22 ± 8.70
24 h	173.95 ± 4.72

**Table 4:** Compounds present in Castilla Rose plant before solid-state fermentation.

ID	Retention Time	Mass	Compound	Family
1	2.15	481.1	HHDP-hexoxide	Ellagitannins
2	8.27	577.1	Procyanidindimer B1	Proanthocyanidin dimers
3	13.01	577.1	Pelargodinin 3-o-rutinoside	Proanthocyanidin dimers
4	14.17	865.3	Procyanidintrimer C1	Proanthocyanidins trimers
5	19.42	289.1	(+)-Catechin	Catechinns
6	20.99	625.1	Quercetin 3,4’-O-diglucoside	Flavonols
7	26.36	595.1	Quercetin 3-O-glucosilxiloside	Flavonols
8	27.53	301.1	Ellagicacid	Hydroxybenzoic acid dimers
9	29.52	463.1	Ellagicacidglucoside	Hydroxybenzoic acid dimers
10	32.22	507.1	Delphinidin 3-O-(6 acetyl-glucoside)	Anthocyanins

**Table 5:** Identified compounds from the RP-HPLC-ESI-MS of the solid-state fermentation of Castilla Rose.

ID	Retention Time	Mass	Compound	Family
1	3.59	480.8	HHDP-hexoxide	Ellagitannins
2	6.77	752.7	1,2-Disinapoylgentibiose	Methoxycinnamic acids
3	8.48	752.7	1,2-Disinapoylgentibiose	Methoxycinnamic acids
4	14.6	770.7	Kaempferol 3,7,4’-O-triglucoside	Flavonols
5	15.74	752.7	1,2-Disinapoylgentiobiose	Methoxycinnamic acids
6	19.28	770.7	Kaempferol 3-O-sophoroside 7-O-glucoside	Flavonols
7	20.55	770.7	Quercetin 3-O-glucosyl-ramnosyl-galactoside	Flavonols
8	21.98	576.7	Procyanidindimer B1	Proanthocyanindin dimers
9	22.7	577	Procyanidindimer B2	Proanthocyanidin dimers
10	23.6	288.7	(+)-Catechin	Catechins
11	24.49	608.7	Kaempferol 3,7-O-diglucoside	Flavonols
12	25.46	576.7	Procyanidindimer B3	Proanthocyanidin dimers
13	26.69	864.6	Procyanidintrimer C1	Proanthocyanidins trimers
14	27.19	288.7	(−)-Epicatequin	Catechins
15	28.76	864.6	Procyanidintrimer C1	Proanthocyanidin trimers
16	29.39	782.6	Terflavin B	Ellagitannins
17	30.49	624.7	Quercetin 3,4’-O-diglucoside	Flavonols
18	32.06	300.6	Ellagicacid	Hydroxybenzoic acid dimers
19	35.54	506.7	Delphinidin 3-O-(6’’-acetyl-glucoside)	Anthocyanins
20	40.95	330.6	Gallicacid 4-O-glucoside	Hydroxybenzoic acids
21	42.6	562.9	Apigeninarabinoside-glucoside	Flavones
22	44.86	326.8	p-Coumaricacid 4-O-glucoside	Hydroxycinnamic acids
23	45.73	344.6	Rosmanol	Phenolicterpenes
24	46.39	560.9	Vitisin A	Polymeric anthocyanins
25	47.88	324.8	p-Coumaroyltyrosine	Hydroxycinnamicacids

**Table 6:** Results of the antioxidant activities tested.

Antioxidant Assay	Inhibition (%)
ABTS	94.34 ± 1.98
DPPH	68.71 ± 0.97
Lipid oxidation	71.49 ± 1.25

## References

[1] Mountain States Wholesale Nursery. https://mswn.com/wp-content/uploads/info-sheets/Purshia-plicata.pdf.

[2] Sepúlveda L., De la Cruz R., Buenrostro J.J., Ascacio-Valdés J.A., Aguilera-Carbó A.F., Prado A., Rodríguez-Herrera R., Aguilar C.N. (2016). Effect of different polyphenol sources on the efficiency of ellagic acid release by *Aspergillus niger*. Rev. Argent. Microbiol..

[3] Quideau S., Deffieux D., Douat-Casassus C., Pouységu L. (2011). Plant polyphenols: Chemical properties, biological activities, and synthesis. Angew. Chem. Int. Ed..

[4] Torres-León C., Ventura-Sobrevilla J., Serna-Cock L., Ascacio-Valdés J.A., Contreras-Esquivel J., Aguilar C.N. (2017). Pentagalloylglucose (PGG): A valuable phenolic compound with functional properties. J. Funct. Foods.

[5] Khanbabaee K., Ree T. (2001). van Tannins: Classification and Definition. Nat. Prod. Rep..

[6] Vázquez Flores A.A., Álvarez Parrilla E., López Díaz J.A., Wall Medrano A., De la Rosa L.A. (2012). Taninos hidrolizables y condensados: Naturaleza química, ventajas y desventajas de su consumo. Tecnociencia Chihuahua.

[7] Aguilera-Carbo A.F., Augur C., Prado-Barragan L.A., Aguilar C.N., Favela-Torres E. (2008). Extraction and analysis of ellagic acid from novel complex sources. Chem. Pap..

[8] Alberto M.R., Rinsdahl Canasovio M.A., Manca de Nadra M.C. (2006). Antimicrobial effect of polyphenols from apple skins on human bacterial pathogens. Electron. J. Biotechnol..

[9] Burboa E.A., Ascacio-Valdés J.A., Zugasti-Cruz A., Rodríguez-Herrera R., Aguilar C.N. (2014). Antioxidant and antibacterial capacity of candelilla extracts residues. Rev. Mex. Ciencias Farm..

[10] Hope Smith S., Tate P.L., Huang G., Magee J.B., Meepagala K.M., Wedge D.E., Larcom L.L. (2004). Antimutagenic Activity of Berry Extracts. J. Med. Food.

[11] Domínguez-Rodríguez G., Marina M.L., Plaza M. (2017). Strategies for the extraction and analysis of non-extractable polyphenols from plants. J. Chromatogr. A.

[12] Pandey A. (2003). Solid-state fermentation. Biochem. Eng. J..

[13] Aguilar C.N., Aguilera-Carbo A., Robledo A., Ventura J., Belmares R., Martinez D., Rodríguez-Herrera R., Contreras J. (2008). Production of antioxidant nutraceutlcals by solid-state cultures of pomegranate (*Punica granatum*) peel and creosote bush (*Larrea tridentata*) leaves. Food Technol. Biotechnol..

[14] Hernández C., Ascacio-Valdés J., De la Garza H., Wong-Paz J., Aguilar C.N., Martínez-Ávila G.C., Castro-López C., Aguilera-Carbó A. (2017). Polyphenolic content, in vitro antioxidant activity and chemical composition of extract from *Nephelium lappaceum* L. (Mexican rambutan) husk. Asian Pac. J. Trop. Med..

[15] Swain T., Hillis W.E. (1959). The phenolic constituents of Prunus domestica. I.—The quantitative analysis of phenolic constituents. J. Sci. Food Agric..

[16] Ascacio-Valdés J.A., Aguilera-Carbó A., Martínez-Hernández J.L., Rodríguez-Herrera R., Aguilar C.N. (2010). *Euphorbia antisyphilitica* residues as a new source of ellagic acid. Chem. Pap..

[17] Ascacio-Valdés J.A., Aguilera-Carbó A.F., Buenrostro J.J., Prado-Barragán A., Rodríguez-Herrera R., Aguilar C.N. (2016). The complete biodegradation pathway of ellagitannins by *Aspergillus niger* in solid-state fermentation. J. Basic Microbiol..

[18] Re R., Pellegrini N., Proteggente A., Pannala A., Yang M., Rice-Evans C. (1999). Antioxidant activity applying an improved ABTS radical cation decolorization assay. Free Radic. Biol. Med..

[19] Meléndez Norma P., Nevárez-Moorillón V., Rodríguez-Herrera R., Espinoza José C., Aguilar C.N. (2014). A microassay for quantification of 2,2-diphenyl-1-picrylhydracyl (DPPH) free radical scavenging. Afr. J. Biochem. Res..

[20] Mendez-Flores A., Hérnandez-Almanza A., Sáenz-Galindo A., Morlett-Chávez J., Aguilar C.N., Ascacio-Valdés J. (2018). Ultrasound-assisted extraction of antioxidant polyphenolic compounds from *Nephelium lappaceum* L. (Mexican variety) husk. Asian Pac. J. Trop. Med..

[21] Mussatto S.I., Aguilar C.N., Rodrigues L.R., Teixeira J.A. (2009). Colonization of *Aspergillus japonicus* on synthetic materials and application to the production of fructooligosaccharides. Carbohydr. Res..

[22] Robledo A., Aguilera-Carbó A., Rodriguez R., Martinez J.L., Garza Y., Aguilar C.N. (2008). Ellagic acid production by *Aspergillus niger* in solid state fermentation of pomegranate residues. J. Ind. Microbiol. Biotechnol..

[23] Gowthaman M.K., Krishna C., Moo-Young M. (2001). Fungal solid state fermentation—An overview. Appl. Mycol. Biotechnol..

[24] Pandey A. (1991). Effect of particle size of substrate of enzyme production in solid-state fermentation. Bioresour. Technol..

[25] Buenrostro-Figueroa J., Ascacio-Valdés A., Sepúlveda L., De La Cruz R., Prado-Barragán A., Aguilar-González M.A., Rodríguez R., Aguilar C.N. (2014). Potential use of different agroindustrial by-products assupports for fungal ellagitannase production undersolid-state fermentation. Food Bioprod. Process..

[26] Krishna C. (2005). Solid-State Fermentation Systems—An Overview. Crit. Rev. Biotechnol..

[27] Graminha E.B.N., Gonçalves A.Z.L., Pirota R.D.P.B., Balsalobre M.A.A., Da Silva R., Gomes E. (2008). Enzyme production by solid-state fermentation: Application to animal nutrition. Anim. Feed Sci. Technol..

[28] Cruz-Hernández M., Contreras-Esquivel J.C., Lara F., Rodríguez R., Aguilar C.N. (2005). Isolation and evaluation of tannin-degrading fungal strains from the Mexican desert. Z. Naturforsch. Sect. C J. Biosci..

[29] Martins S., Mussatto S.I., Martínez-avila G., Montañez-saenz J., Aguilar C.N., Teixeira J.A. (2011). Bioactive phenolic compounds: Production and extraction by solid-state fermentation. A review. Biotechnol. Adv..

[30] Sepúlveda L., Aguilera-Carbó A., Ascacio-Valdés J.A., Rodríguez-Herrera R., Martínez-Hernández J.L., Aguilar C.N. (2012). Optimization of ellagic acid accumulation by *Aspergillus niger* GH1 in solid state culture using pomegranate shell powder as a support. Process Biochem..

[31] Yadav J.S. (1988). SSF of wheat straw with alcaliphilic Coprinus. Biotechnol. Bioeng..

[32] Martins S., Teixeira J.A., Mussatto S.I. (2013). Solid-state fermentation as a strategy to improve the bioactive compounds recovery from larrea tridentata leaves. Appl. Biochem. Biotechnol..

[33] Barr D.P., Aust S.D. (1994). Pollutant degradation by white rot fungi. Rev. Environ. Contam. Toxicol..

[34] Schofield P., Mbugua D.M., Pell A.N. (2001). Analysis of condensed tannins: A review. Anim. Feed Sci. Technol..

[35] Aguilar-Zarate P., Wong-Paz J.E., Buenrostro-Figueroa J.J., Ascacio J.A., Contreras-Esquivel J.C., Aguilar C.N. (2017). Ellagitannins: Bioavailability, Purification and Biotechnological Degradation. Mini Rev. Med. Chem..

[36] Ito H., Miyake M., Nishitani E., Mori K., Hatano T., Okuda T., Konoshima T., Takasaki M., Kozuka M., Mukainaka T. (1999). Anti-tumor promoting activity of polyphenols from *Cowania mexicana* and *Coleogyne ramosissima*. Cancer Lett..

[37] Sepúlveda L., Ascacio A., Rodríguez-Herrera R., Aguilera-Carbó A., Aguilar C.N. (2011). Ellagic acid: Biological properties and biotechnological development for production processes. Afr. J. Biotechnol..

[38] Kashyap D., Sharma A., Tuli H.S., Sak K., Punia S., Mukherjee T.K. (2017). Kaempferol—A dietary anticancer molecule with multiple mechanisms of action: Recent trends and advancements. J. Funct. Foods.

[39] Pandima K., Sheeja D., Fazel S., Sureda A., Xiao J., Mohammad S., Daglia M. (2015). Kaempferol and inflammation: From chemistry to medicine. Pharmacol. Res..

[40] Fraga C.G., Oteiza P.I. (2011). Free Radical Biology & Medicine Dietary fl avonoids: Role of (−)-epicatechin and related procyanidins in cell signaling. Free Radic. Biol. Med..

[41] Fernández-Larrea J., Pinent M., Bladé M.C., Salvadó M.J., Blay M., Pujadas G., Ardévol A., Arola L. (2007). Alimentos ricos en procianidinas, alimentatión funcional para prevenir la aparición de síndrome metabólico. Rev. Esp. Obes..

[42] Byun E., Sung N., Byun E., Song D., Kim J., Park J., Song B., Park S., Lee J., Byun M. (2013). The procyanidin trimer C1 inhibits LPS-induced MAPK and NF- κ B signaling through TLR4 in macrophages. Int. Immunopharmacol..

[43] Mesa-Vanegas A.M., Gaviria C.A., Cardona F., Sáez-Vega J.A., Trujillo S.B., Rojano B.A. (2010). Antioxidant activity and total phenols content from some species of *Calophyllum* genus. Rev. Cuba. Plantas Med..

[44] Deng Y.T., Liang G., Shi Y., Li H.L., Zhang J., Mao X.M., Fu Q.R., Peng W.X., Chen Q.X., Shen D.Y. (2016). Condensed tannins from Ficus altissima leaves: Structural, antioxidant, and antityrosinase properties. Process Biochem..

[45] Aguirre-Joya J.A., Pastrana-Castro L., Nieto-Oropeza D., Ventura-Sobrevilla J., Rojas-Molina R., Aguilar C.N. (2018). The physicochemical, antifungal and antioxidant properties of a mixed polyphenol based bioactive film. Heliyon.

[46] Tan J.B.L., Lim Y.Y. (2015). Critical analysis of current methods for assessing the in vitro antioxidant and antibacterial activity of plant extracts. Food Chem..

[47] Prior R.L., Wu X., Schaich K. (2005). Standardized methods for the determination of antioxidant capacity and phenolics in foods and dietary supplements. J. Agric. Food Chem..

